# Enlargement of the Field of View and Maintenance of a High Signal-to-Noise Ratio Using a Two-Element High-T_c_ Superconducting Array in a 3T MRI

**DOI:** 10.1371/journal.pone.0042509

**Published:** 2012-08-03

**Authors:** In-Tsang Lin, Hong-Chang Yang, Jyh-Horng Chen

**Affiliations:** 1 Interdisciplinary MRI/MRS Lab, Department of Electrical Engineering, National Taiwan University, Taipei, Taiwan; 2 Department of Physics, National Taiwan University, Taipei, Taiwan; The University of Chicago, United States of America

## Abstract

This study examines the enlargement of the field of view (FOV) and the maintenance of a high signal-to-noise ratio (SNR) through the use of two high-temperature superconducting (HTS) resonators in a 3T MRI. Two Bi_2_Sr_2_Ca_2_Cu_3_O_x_ (Bi-2223) surface resonators, each of 4-cm diameter, were used in a 3T MRI. Professionally made copper resonators operate at 300 K, but each Bi-2223 resonator, operated at 77 K and demonstrated a 3.75 fold increase in SNR gain. For the same scanning time, the SNR of the images of a rat’s brain and back, obtained using two small Bi-2223 surface resonators, was higher than that obtained using a single 8-cm surface resonator.

## Introduction

Magnetic Resonance Imaging (MRI) has become widespread in biomedical research. The MRI image is correlated with the signal-to-noise ratio (SNR) of the magnetic resonance (MR) signals induced in the imaging resonators. These MR signals are weak, because of the small difference in the proton populations of the parallel and anti-parallel spins (∼18 ppm at 3T for protons) that contribute to the signal [1]. To improve SNR in MR imaging, many studies have attempted to increase the sensitivity of the resonator by replacing the conventional copper-receiving resonator with either a high-temperature superconducting (HTS) thin-film resonator [2], [3], [4], a HTS surface resonator [5], or a cryogenic probe [6]. In recent years, relatively inexpensive bismuth-based HTS tapes, such as Bi_2_Sr_2_Ca_2_Cu_2_O_3_ (Bi-2223) and Bi_2_Sr_2_Ca_1_Cu_2_O_3_ (Bi-2212) tapes, have demonstrated increased potential for use in radio frequency (RF) surface resonators in MRI. Grasso *et al.* [7] measured the quality factor (QF) of HTS tape surface resonators made using Bi-2223 tapes and observed that the QF values were four times higher than those of commercially available MRI resonators, at 8 MHz. Jing *et al.* [8] theoretically measured the QF value of Bi-2223 surface resonators and verified the performance of Bi-2223 surface resonators by experiment. These two studies reported that Bi-2223 surface resonators can yield a higher SNR than copper surface resonators.

However, one of the limitations of using a Bi-2223 surface resonator for MRI is the small field of view (FOV). During the imaging process, the region of interest (ROI) is often not known, prior to the first scan. Therefore either a large resonator must be used to obtain a large FOV, or there is a risk of having to reposition a smaller resonator and repeat the scan. This problem occurs often when imaging a larger area, when repositioning the resonator can be time consuming. There are two methods of increasing the FOV of the resonator. One solution uses multiple surface resonators connected to multiple receiver channels [9], but this is an expensive solution, because of the high cost of the additional receiver channels. The second method increases the area being imaged by using multiple resonators, which are arranged to minimize their mutual inductances [10]. The method for remotely selecting the sensitive region of a surface resonator uses mutually-coupled resonator arrays (MCRA). No studies have reported on the feasibility of using liquid nitrogen-cooled Bi-2223 surface MCRA at 3T. This study uses the latter method of improving the FOV by using HTS MCRA. This paper investigates the enlargement of the FOV and maintenance of a high SNR through the use of two 4-cm Bi-2223 surface resonators, instead of a single 8-cm HTS surface resonator. These arrays consist of two HTS primary receiver resonators inductively coupled to a single secondary resonator. The use of HTS MCRA for the enlargement of the FOV and the maintenance of a high SNR are investigated in this study.

## Results

### MR Images of a Phantom

The unloaded and loaded QF values for the 4-cm surface resonator of the HTS MCRA, at 77 K, were 1330 and 985, respectively. Similarly, the unloaded and loaded QF values of the 4-cm professionally made resonator of the copper MCRA, at 300 K, were 300 and 155, respectively. The unloaded and loaded QF values of the 4-cm professionally made resonator of the copper MCRA, at 77 K, were 578 and 350, respectively. The predicated SNR gain for the resonator of the HTS MCRA at 77 K was 3.76 and 1.85 times higher than those for the resonator of the copper MCRA at 300 K and at 77 K, respectively.

**Figure 1:**
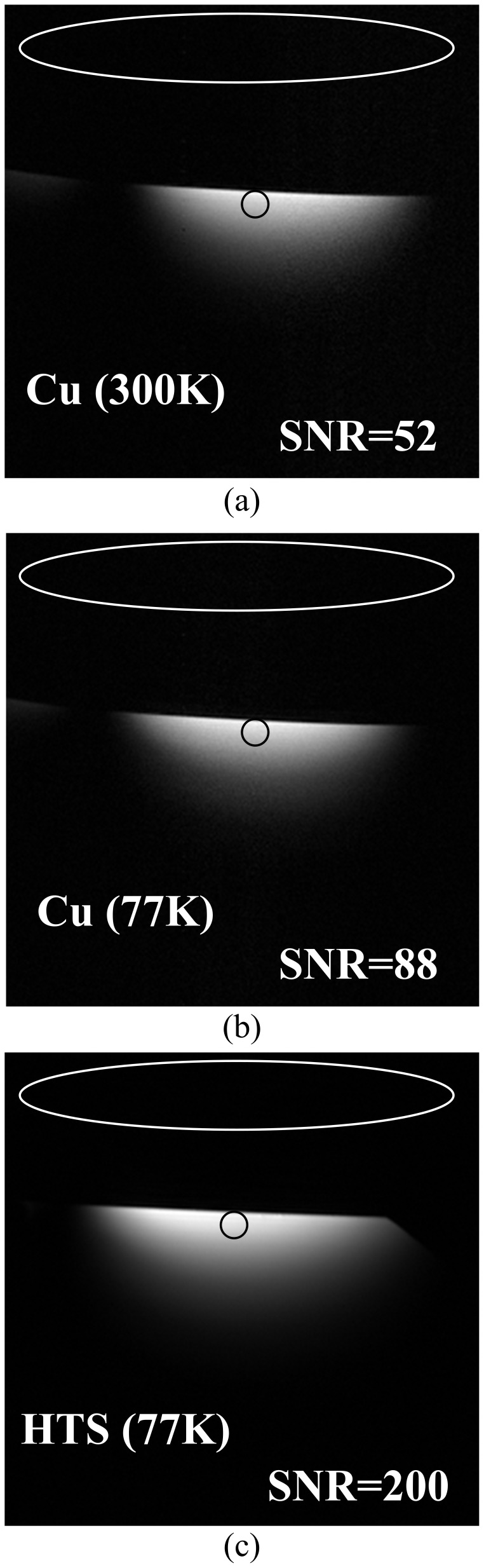
Phantom images were acquired using (a) Copper resonator at 300 K with SNR = 52, (b) Copper resonator at 77 K with SNR = 88 and (c) HTS resonator at 77 K with SNR = 200.

The images were used to evaluate the performance of the resonator of the HTS MCRA and the resonator of the copper MCRA. A comparison of the coronal phantom images obtained using the resonator of the HTS and the copper MCRA is shown in Figure 1. Figures 1(a) and 1(b) show the image acquired using the resonator of the copper MCRA, at 300 K and at 77 K, while Figure 1(c) shows the image acquired using the resonator of the HTS MCRA, at 77 K. In the pictures in Figure 1, the black circle represents the mean of ROI and the white circle represents the standard deviation (STD). The SNR of the resonator of the HTS MCRA at 77 K was 200, which is 3.84 times higher than the value of 52, which was obtained when using the resonator of the copper MCRA at 300 K.

### The Relationship between Measured SNR and Depth

**Figure 2:**
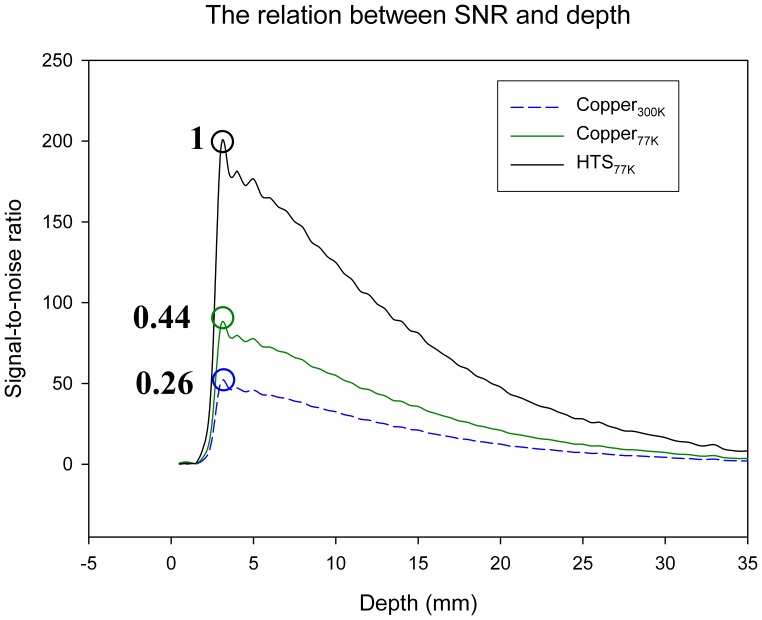
The experimental data used to determine the relationship between SNR and depth.

When the phantom images had been acquired, the performance of each RF resonator could be compared quantitatively, with respect to the relationship between SNR and depth. The resonator of the HTS MCRA at 77 K, the resonator of the copper MCRA at 300 K and the resonator of the copper MCRA at 77 K were individually placed in the cryostat, in order to measure the SNR. The results are shown in Figure 2, which illustrates the experimentally determined relationship between SNR and depth. The x-axis represents the depth. The RF resonator plane is defined as zero depth. The y-axis represents the SNR. The distance between the RF resonator plane and the surface of the phantom is 2 mm. Figure 2 shows that the maximum value is obtained for the resonator of the HTS MCRA at 77 K; the minimum value is obtained for the resonator of the copper MCRA at 300 K. The black and blue circles show that the resonator of the HTS MCRA at 77 K is superior to the resonator of the copper MCRA at 300 K, providing about 3.84 times the gain in SNR value. The black and green circles show that the resonator of the HTS MCRA at 77 K is superior to the resonator of the copper MCRA at 77 K, providing about 2.27 times the gain in SNR value.

Figure 2 shows that the value for SNR using the resonator of the HTS MCRA at 77 K is equal to 50, when the depth is 20 mm and the value for SNR using the resonator of the copper MCRA at 300 K is equal to 10, when the depth is 20 mm. It is also seen that the signal attenuation in the resonator of the copper MCRA is larger than that in the resonator of the HTS MCRA.

### Rat Images Using Dual Bi-2223 Resonators

**Figure 3:**
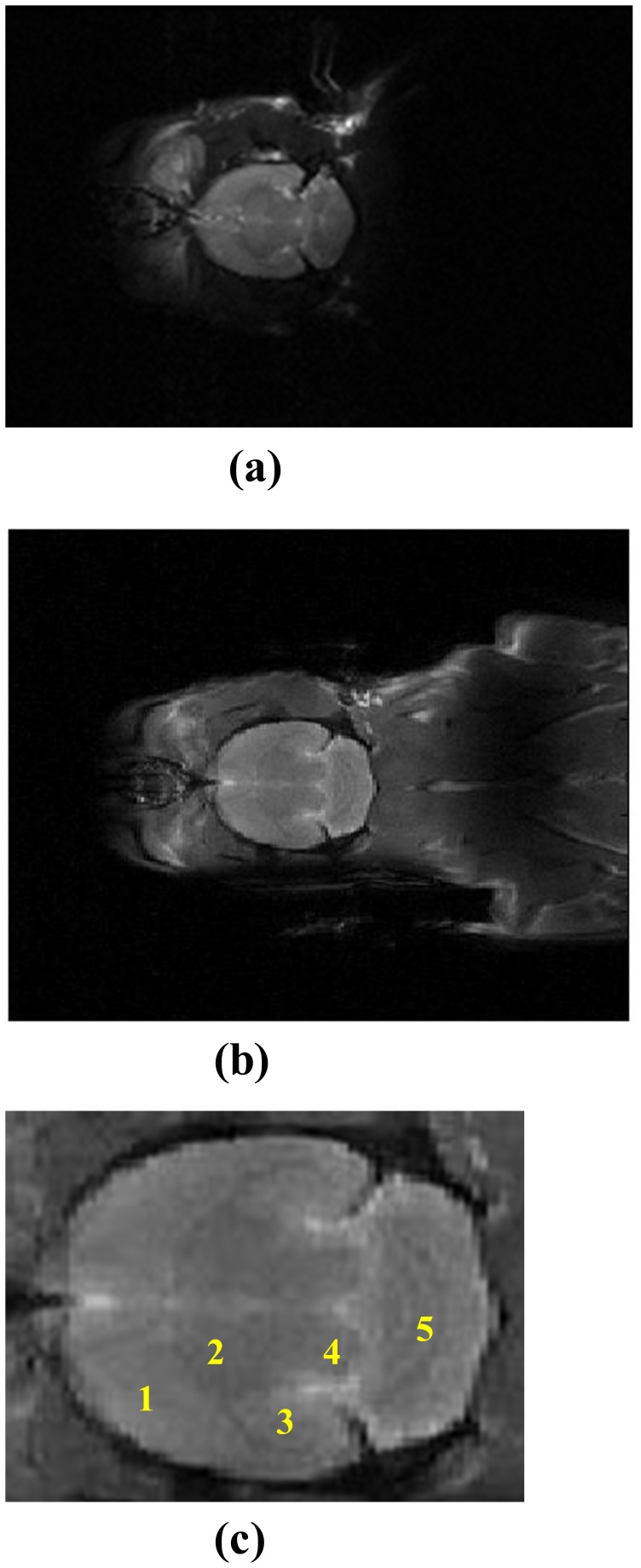
Images of the brain and body of a rat using (a) the copper surface resonator at 300 K, (b) the HTS tape surface resonator at 77 K and (c) 3X magnification of the rat brain portion of Fig. 3(b), showing details of the rat brain.

Further experiments were performed, involving rat brain and body imaging. Two HTS surface resonators were employed to increase the length of the scanned image. Figure 3(a) shows the axial view of an *in-vivo* rat image, acquired using a copper resonator at 300 K. Figure 3(b) shows the axial view of an *in-vivo* rat image, acquired using two HTS surface resonators at 77 K. Figures 3(a) and 3(b) show that the length of the image is enlarged by the simultaneous use of two HTS surface resonators for the scan. Figure 3(c) shows a 3X magnification of the rat brain portion of Figure 3(b), showing details of the rat brain with clear labels. The structure in Figure 3(c) shows (1) caudate putamen, (2) thalamus, (3) hippocampus, (4) mesencephalon and (5) cerebral lobule.

## Discussion

Hyde *et al.* first proposed increasing the effective FOV in high-resolution ^1^H imaging by switching between multiple surface-resonator receivers, arranged to minimize their mutual inductance [11]. Roemer *et al.* later demonstrated a system using four receiver coils and four receiver channels, calling it an NMR phased array [9]. Additionally, Roemer described methods for combining the data from the multiple receivers, in order to optimize the SNR in every voxel. The decoupling method uses overlapping adjacent resonators and attaches low input impedance preamplifiers to all resonators. Wright et al. proposed another method to increase the area being imaged by multiple resonators, which are specially arranged to minimize their mutual inductances [10]. The method uses MCRA, which are particularly well suited for use as switchable resonators. Only one element of the resonator array is directly connected to the receiver, which allows flexibility in the system design and implementation. For example, multiple primary coils can be resonated simultaneously, in order to allow both the size and the location of the sensitive region to be changed. Because coils that are not selected are detuned and all mutual coupling effects between selected coils are included in the analysis, primary coils need not be isolated (no mutual coupling) from each other. The system of three resonators, used in this study, includes two primary resonators and one secondary resonator; only one secondary resonator of the resonator array is directly connected to the receiver. The method of coupling is identical to Wright’s coupling method.

Another issue is the depth of RF penetration into the HTS phased array. Yoshioka *et al.* showed that a small surface resonator of 23 mm is superior to a large surface resonator of 80 mm [12]. Small surface resonators usually have a higher SNR, because they receive noise only from nearby regions. This study uses two (4 cm) HTS surface resonators to improve the FOV, instead of one large (8 cm) HTS surface resonator. However, the two 4-cm high T_c_ superconducting resonators result in reduced RF penetration in a rat, because of the smaller size of the resonator. Combined with the array construction, the two HTS resonators expand the FOV to beyond that of an individual HTS resonator, while maintaining a high SNR gain, because of the small size of the resonator.

The authors previously studied a Bi-2223 surface resonator of 4-cm diameter [13]. Using a Bi-2223 surface resonator at 77 K a SNR for rat brain imaging showed a gain of 3.75 over a similar copper resonator at 300 K. However, the small FOV is a limitation, when using a Bi-2223 surface resonator for MRI. Hence, a new method is proposed, which simultaneously uses two Bi-2223 surface resonators for 3T. All results are based on one dataset. The SNR gain for HTS MCRA over copper MCRA was observed in the *in vivo* rat brain, which is similar to that for a single surface resonator configuration. This study demonstrates the advantages of using two HTS surface resonators in a Bruker 3T MR scanner. The advantages of using an array of small surface resonators, instead of a larger resonator with the same FOV, are that (i) there is no increase in imaging time; this conclusion is also noted by Roemer *et al.* [9] (ii) there is a higher SNR; this conclusion is also noted by Yoshioka *et al.* [12], and (iii) there is improved sensitivity at all depths; this conclusion is also noted by Wright *et al* [14].

A related study of HTS arrays was completed by Wosik *et al.*, who reported on a planar 200 MHz superconducting two-resonator array for magnetic resonance imaging applications [15]. The array was constructed from a double-sided, thin YBa_2_Cu_3_O_7−x_ film on r-cut sapphire substrate and consisted of two 24 mm diameter resonators with built-in planar capacitors for coupling, tuning and matching electronics. Wosik *et al.* developed a HTS-based, multilayered planar structure (HTS/dielectric/metal), which, in addition to HTS resonators, included built-in resonator capacitors for tuning, matching and coil-to-coil decoupling. This study uses a different HTS material: Bi-2223 tape. Wosik’s decoupling method uses built-in resonator capacitors for tuning, matching and coil-to-coil decoupling, but this study uses a MCRA for decoupling.

This is the first study to integrate two HTS surface resonators in order to enhance the SNR and enlarge the FOV in a 3T MRI. Consistent with previous reports related to cooled MRI surface resonators [16], [17], [18], [19], [20], [21], the results show promising evidence that a HTS surface resonator is a useful imaging component for MRI studies, especially for animal studies. In this study, *in-vivo* animal imaging also showed that two HTS resonators at 77 K produced significant SNR gains of 3.75, compared with the copper resonator at 300 K. Combined with the array construction, the two HTS resonators expand the FOV to beyond that of an individual HTS resonator, while maintaining a high SNR gain, because of the small size of the resonator. Imaging results are shown in Figure 3(b). The dark MRI signal region near the rat’s shoulder in Figure 3(b) is due to some B_1_ cancellation of the signals for two HTS resonators. The enlarged FOV reduces the scanning time for the whole body imaging of a rat. Because of its ease of implementation and the flexibility of the design, HTS mutually coupled arrays should be of use in a number of applications in MR imaging, such as the design of resonators for multinuclear study [22]. In the future, it should be possible to use four HTS resonators in a newly designed resonator [23]. The relationship between resonator size, resonator-to-body distance and SNR will be the next area of study. Most importantly, two HTS resonators may be combined with parallel imaging techniques, to save imaging time, and may also be used in MR microscopy for small animals and for human pathology modeling, which often demands high resonator sensitivity.

## Materials and Methods

### Evaluation of SNR Gain Using the Quality Factor

For small samples, the noise from the resistance of the receiving surface resonator may dominate the SNR of MR signals. Therefore, reducing the resistance of the surface resonator effectively increases the SNR. According to the derivation by Hoult and Richards [24], the relationship between SNR and surface resonator factors can be formulated as follows,


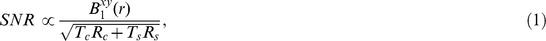


where *B_1_^xy^* represents the magnetic field produced by the unit current, *T*_c_ and *T*_s_ are the temperatures of the surface resonator and the sample, respectively, and *R_c_* and *R_s_* are the resistances of the surface resonator and the sample, respectively.

The improvement in SNR for the use of HTS resonators, instead of room-temperature copper resonators, can be determined using QF characterization. Inductive measurement techniques can be used to determine the quality factor of HTS resonators under different loading conditions. The loaded and unloaded QF values allow accurate estimation of the respective contributions to the induced sample and internal resonator losses to the SNR. The sensitivity factor for unloaded HTS resonators can be measured directly using a single-loop probe method [25]. In this study, QF values under unloaded and loaded conditions were measured using a standard reflectometry technique, whereby the resonator to be tested is matched to a 50 Ω resistance, via an inductive copper matching-loop. The ratio of loaded to unloaded QF values can is used to evaluate the loaded sensitivity factor, using equation (2).


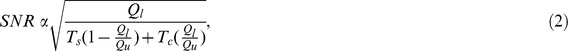


where *Q_l_* is the loaded QF value, *Q_u_* is the unloaded QF value, *T_c_* is the temperature of the copper or the HTS resonator and *T_s_* is the temperature of sample. From the material aspect, it should be noted that a fair comparison between a HTS resonator and a copper resonator requires the same design and geometrical parameters for both resonators.

### Hardware

**Figure 4:**
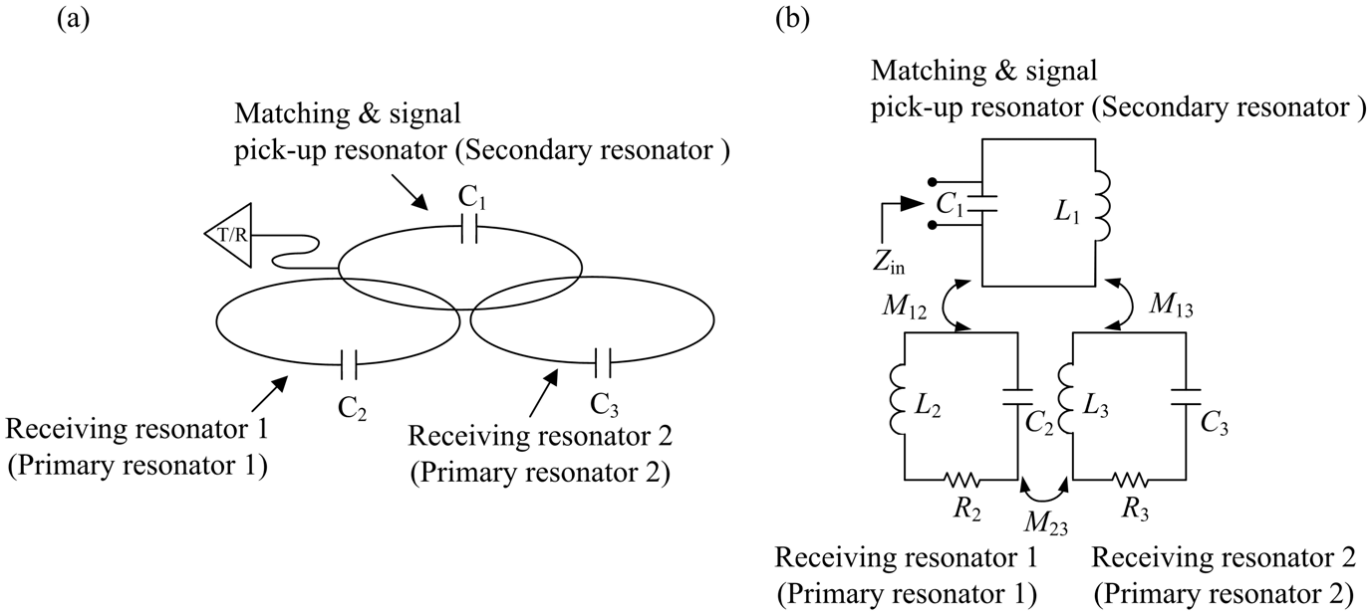
(a) Illustration of the operation of mutually coupled resonators. The primary receiver resonator acts as a standard local resonator, receiving energy from excited spins. Current is generated in the primary resonator by an EMF at the terminals of the secondary resonator. (b) The equivalent circuit of the inductively coupled design shows that the RF signal is picked up by mutual inductive coupling.

MR experiments were performed on a Bruker Biospec 3T MRI system (Bruker Biospin, Ettlingen, Germany) with an inserted gradient, for which the maximum gradient strength was 200 mT/m and the inner diameter was 12 cm. The resonator system (Figure 4(a)) consisted of two HTS resonators, receiving resonator 1 (primary resonator 1) and receiving resonator 2 (primary resonator 2). Each HTS resonator comprised a 4-cm circular loop, made from the Bi_2_Sr_2_Ca_2_Cu_3_O_10_/Ag (Bi-2223) tape (Innova Corp., Beijing, China). The high-QF capacitor (22 pF and QF≈1000 at 125 MHz, American Technical Ceramics, NY, USA) was nonmagnetic and was directly soldered at both ends of each tape.

RF signal transmission and reception was accomplished using inductive coupling [26], as shown in Figure 4(b). *M*_12_, *M*_13_ and *M*_23_ are the coupling coefficients of the signal pick-up resonator (secondary resonator) and receiving resonator 1, the signal pick-up resonator and receiving resonator 2 and receiving resonator 1 and receiving resonator 2, respectively. If the distance between the signal pick-up resonator and receiving resonator 1 decreases, then *M*_12_ increases. Therefore, the distances between these three resonators must be repeatedly modified, by trial and error. Note that most of the protocols used images of small animals, requiring transmit/receive (T_x_/R_x_) separation. However, the T_x_/R_x_ configuration was used, because of the space limitations of the gradient bore. The cryostat was placed inside the 12-cm diameter gradient bore, leaving no space for the volume coil as a transmitter. In order to perform the HTS experiment, the cryostat and the T_x_/R_x_ configuration were required, although the T_x_/R_x_ configuration reduces the SNR.

**Figure 5:**
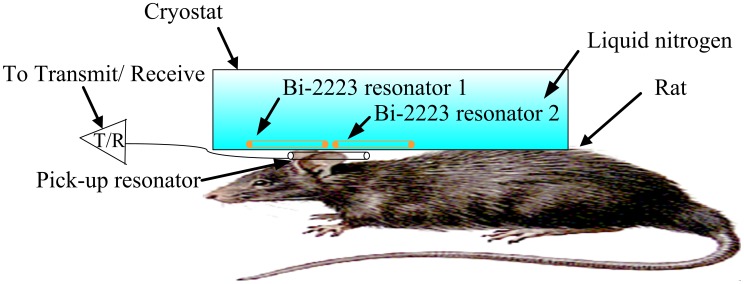
The 3T system setup for the rat experiment, wherein the matching and signal pick-up resonator with a variably tunable capacitance is placed between the two Bi-2223 resonators.

Figure 5 shows the experimental setup, wherein the matching and signal pick-up resonator with a tunable variable capacitance is positioned between the two HTS surface resonators and the rat. The pickup resonator is a 4-cm-diameter circular surface resonator, made from copper. It was placed below the resonator system and outside the cryostat, at 300 K, approximately 2 mm away from the two HTS surface resonators, as shown in Figure 5. One trimmer capacitor (Voltronics Corp., NJ, USA) was soldered to the pick-up surface resonator. Tuning, matching and signal pick-up were achieved by adjusting the relative position of the signal pick-up surface resonator and by tuning a variable trimmer capacitor to cancel out the imaginary part of Z_in_. The same geometry and position were used for both the HTS and professionally made copper resonators.

The professional copper resonator was constructed using copper cable (Hitachi, Japan), with 99.9999% (6N) purity copper cable and a high-Q capacitor (22 pF and Qs≈1000 at 125 MHz, American Technical Ceramics, NY, USA). It was nonmagnetic and was directly soldered at both ends of each cable. The diameter of the copper cable was 1.5 mm.

The cryostat used for rat experiments was placed inside a gradient bore of 12-cm diameter. The cryostat was completely constructed from Styrofoam, to ensure thermal insulation. The two HTS resonators were cooled in a self-designed polystyrene container to 77 K, using liquid nitrogen. The container could maintain a temperature of 77 K for about 1 hour. All of the S-parameter measurements were performed using a network analyzer HP8751A (Agilent, CA, USA). The Q-values of both the HTS and the copper surface resonators were measured under unloaded and loaded conditions. A whole rat was used as the test sample for measurement of the loaded *Q*-factor. The decoupling behaviour in the unloaded and loaded cases is different, because of the coupling effect of the imaging sample. To cure the coupling effect of the imaging sample, the signal pick-up was performed by adjusting the relative position of the signal pick-up surface resonator and by tuning a variable trimmer capacitor to cancel out the imaginary part of Z_in_. When the load under the coil is changed, fine-tuning is required for better decoupling.

The phantom was a cylindrical phantom, 29 mm in diameter and 90 mm in length, filled with 20-mM CuSO_4_ solution. The power setting for each individual resonator was determined by stepping through a range of transmitting attenuation values, in the pre-scans. The Sprague Dawley (SD) adult female rat (weight 250∼300 g) was placed on a heated water pad, to maintain a rectal temperature of ∼37°C when it was in the MRI room. The rat was initially anesthetized with 3% isoflurane in a 1/1 Oxygen/Air mixture and injected with atropine (20 µg/kg), in order to avoid excessive salivation. Each animal was secured in a head holder, with ear bars and a bite bar to prevent head movement, and was strapped to a plastic cradle. Rectal temperature and heart rate were continuously monitored throughout the experiment. The Institutional Animal Care and Use Committee at National Taiwan University approved all of the procedures for the animal experiments.

### Imaging Experiment

Firstly, the performance of both HTS and copper resonators was tested. The images of the phantom were acquired using a fast spin echo sequence with repletion time (TR)/echo time (TE) = 3500/62 ms and NEX  = 1. The FOV, the slice thickness and acquisition matrix sizes were 4 cm×4 cm, 2 mm, and 256×256, respectively. The in-plane resolution was 156 µm. The rat brain and body was imaged using two HTS resonators. All images were acquired using a fast spin echo sequence with TR/TE  = 3500/62 ms and NEX  = 1. Note that the T_1_ value of the rat’s brain at 3T varied from 1084 to 1823 ms. Thus, a TR of 3500 ms was chosen to avoid the saturation effect and to provide a more realistic B_1_ distribution for the resonator [27]. The FOV, slice thickness and acquisition matrix size were 6 cm×8 cm, 2 mm, and 256×256, respectively. The in-plane resolution was 234×312 µm. The optimum transmitted power was set to maximize the signal amplitude of both the HTS and the copper resonators.

Measurement of the SNR for a conventional image uses the mean value of the pixels within the region of interest (ROI), whose size was 0.5 cm×0.5 cm. The standard deviation of the background noise is measured by using the largest possible ROI (avoid ghosting/aliasing or motion artifact regions). The SNR of an image is calculated as the ratio of the mean signal to the standard deviation of the background noise. SNRs were calculated, in order to compare the performance of the HTS and the professionally made copper surface resonators.

## References

[1] Kuo R, Panchal M, Tanenbaum L, Crues III JV (2007) 3.0 Tesla imaging of the musculoskeletal system. Journal of Magnetic Resonance Imaging 25: 245–261. 17260407 10.1002/jmri.20815

[2] Yang H, Tsai K, Chen J, Wu C, Horng H, et al (2007) High-Tc superconducting surface coils for improving the image quality on a 3 T imager. Superconductor Science and Technology 20: 777–780.

[3] Black R, Early T, Roemer P, Mueller O, Mogro-Campero A, et al (1993) A high-temperature superconducting receiver for nuclear magnetic resonance microscopy. Science 259: 793–795. 8430331 10.1126/science.8430331

[4] Ma Q (1999) RF applications of high temperature superconductors in MHz range. IEEE Transactions on Applied Superconductivity 9: 3565–3568.

[5] Okada H, Hasegawa T, Vanheteren J, Kaufman L (1995) RF coil for low-field MRI coated with high-temperature superconductor. Journal of Magnetic Resonance, Series B 107: 158–164.

[6] Wright A, Song H, Wehrli F (2000) In vivo MR micro imaging with conventional radiofrequency coils cooled to 77 K. Magnetic Resonance in Medicine. 43: 163–169. 10.1002/(sici)1522-2594(200002)43:2<163::aid-mrm1>3.0.co;2-k 10680678

[7] Grasso G, Malagoli A, Scati N, Guasconi P, Roncallo S, et al (2000) Radio frequency response of Ag-sheathed (Bi, Pb) 2Sr2Ca2Cu3O10+ x superconducting tapes. Superconductor Science and Technology 13: L15–L18.

[8] Yuan J, Shen G (2004) Quality factor of Bi (2223) high-temperature superconductor tape coils at radio frequency. Superconductor Science and Technology 17: 333–336.

[9] Roemer P, Edelstein W, Hayes C, Souza S, Mueller O (1990) The NMR phased array. Magnetic resonance in medicine 16: 192–225. 2266841 10.1002/mrm.1910160203

[10] Wright SM, Magin RL, Kelton JR (1991) Arrays of mutually coupled receiver coils: theory and application. Magnetic resonance in medicine 17: 252–268. 2067400 10.1002/mrm.1910170128

[11] Hyde JS, Jesmanowicz A, Froncisz W, Kneeland JB, Grist TM, et al (1986) Parallel image acquisition from noninteracting local coils. Journal of magnetic resonance 70: 512–517.

[12] Yoshioka H, Ueno T, Tanaka T, Shindo M, Itai Y (2003) High-resolution MR imaging of triangular fibrocartilage complex (TFCC): comparison of microscopy coils and a conventional small surface coil. Skeletal radiology 32: 575–581. 12942205 10.1007/s00256-003-0672-7

[13] Lin IT, Yang HC, Chen JH (2011) Using high-Tc superconducting resonator for enhancement of diffusion tensor imaging. Journal of Applied Physics 109: 116103.

[14] Wright SM, Wald LL (1997) Theory and application of array coils in MR spectroscopy. NMR in Biomedicine 10: 394–410. 9542737 10.1002/(sici)1099-1492(199712)10:8<394::aid-nbm494>3.0.co;2-0

[15] Wosik J, Xue L, Xie LM, Kamel M, Nesteruk K, et al (2007) Superconducting array for high-field magnetic resonance imaging. Applied Physics Letters 91: 183503–183503–183503.

[16] Neuberger T, Webb A (2008) Radiofrequency coils for magnetic resonance microscopy. NMR Biomed. 10.1002/nbm.1246 18300326

[17] Miller J, Hurlston S, Ma Q, Face D, Kountz D, et al. (1999) Performance of a high-temperature superconducting probe for in vivo microscopy at 2.0 T. Magnetic Resonance in Medicine 41. 10.1002/(sici)1522-2594(199901)41:1<72::aid-mrm11>3.0.co;2-a 10025613

[18] Hurlston S, Brey W, Suddarth S, Johnson G (1999) A high-temperature superconducting Helmholtz probe for microscopy at 9.4 T. Magnetic Resonance in Medicine. 41: 1032–1038. 10.1002/(sici)1522-2594(199905)41:5<1032::aid-mrm23>3.0.co;2-x 10332887

[19] Ginefri J, Poirier-Quinot M, Girard O, Darrasse L (2007) Technical aspects: Development, manufacture and installation of a cryo-cooled HTS coil system for high-resolution in-vivo imaging of the mouse at 1.5 T. Methods. 43: 54–67. 10.1016/j.ymeth.2007.03.011 17720564

[20] Black R, Early T, Roemer P, Mueller O, Mogro-Campero A, et al (1993) A high-temperature superconducting receiver for nuclear magnetic resonance microscopy. Science 259: 793. 8430331 10.1126/science.8430331

[21] Darrasse L, Ginefri J (2003) Perspectives with cryogenic RF probes in biomedical MRI. Biochimie 85: 915–937. 14652180 10.1016/j.biochi.2003.09.016

[22] Keinan-Adamsky K, Shinar H, Navon G (2006) Multinuclear NMR and MRI studies of the maturation of pig articular cartilage. Magnetic Resonance in Medicine 55: 532–540. 16450338 10.1002/mrm.20775

[23] Zhang X, Zhu XH, Chen W (2005) Higher-order harmonic transmission-line RF coil design for MR applications. Magnetic resonance in medicine 53: 1234–1239. 15844152 10.1002/mrm.20462

[24] Hoult DI, Richards RE (1976) The signal-to-noise ratio of the nuclear magnetic resonance experiment. J Magn Reson 24: 71–85. 10.1016/j.jmr.2011.09.018 22152352

[25] Ginefri JC, Durand E, Darrasse L (1999) Quick measurement of nuclear magnetic resonance coil sensitivity with a single-loop probe. Review of Scientific Instruments 70: 4730–4731.

[26] Hoult DI, Tomanek B (2002) Use of Mutually Inductive Coupling in Probe Design. Concepts in Magnetic Resonance 15: 262–285.

[27] Stanisz GJ, Odrobina EE, Pun J, Escaravage M, Graham SJ, et al (2005) T1, T2 relaxation and magnetization transfer in tissue at 3T. Magnetic Resonance in Medicine 54: 507–512. 16086319 10.1002/mrm.20605

